# Pamphlets Received

**Published:** 1879-04

**Authors:** 


PAMPHLETS RECEIVED.—The Therapeutic Value of Ergot. By J. W. Compton, M.D., Professor of Materia Medica and Therapeutics in the Medical College of Evansville, Indiana. Reprint from Detroit *Lancet*, March, 1879.

The Non-Asylum Treatment of the Insane. By Wm. A. Hammond, M.D., Professor of Diseases of the Mind and Nervous System in the Medical Department of the University of the City of New York, etc. 1879.

A Chart of all the Common Poisons, with the prominent symptoms and full directions for treatment carefully compiled from the latest and best authorities, and arranged for instantaneous reference. By J. E. Sanborn. M.D., Rockport, Mass. 1879.

A Concise Epitome of Cutaneous Diseases, according to the latest and most approved classification. By J. E. Sanborn, M.D. Rockport, Mass. 1879.

We are in receipt of the prospectus of the *Journal of Physiology*, which exhibits a splendid corps of contributors and editors. It is an enterprise that supplies a pressing want, and we most earnestly hope it success.

We have received *The Physician's Visiting List* for 1879, published by Messrs. Lindsay & Blakiston, Philadelphia. This is the twenty-eighth year of its publication, and its value has long since been found and appreciated.

---

The following is a list of the graduates in the various Medical Colleges for the past year, so far as heard from up to date:

| | |
|---|---|
| Atlanta Medical College | 35 |
| Bellevue Hospital Medical College | 165 |
| Cincinnati College of Medicine and Surgery | 32 |
| Columbus (Ohio) Medical College | 51 |
| Chicago Medical College | 35 |
| College of Physicians and Surgeons, N. Y | 95 |
| Hospital Medical College, Louisville | 18 |
| Jefferson Medical College | 196 |
| Louisville Medical College | 60 |
| Medical Department of the University of Nashville and Vanderbilt University | 115 |
| Medical College of Ohio | 103 |
| Medical Department University of the City of New York | 205 |
| Medical Department University of Pennsylvania | 91 |
| Missouri Medical College, St. Louis | 88 |
| Medical Department University of Louisville | 84 |
| Medical Department University of Buffalo | 41 |

| | |
|---|---|
| Medical Department University of Iowa | 15 |
| Miami Medical College | 34 |
| Rush Medical College (Chicago) | 121 |
| School of Medicine and Surgery, Maryland | 50 |
| St. Louis Medical College | 58 |
| Woman's Medical College (Chicago) | 5 |
| Yale College Medical School | 8 |

